# SERENA-6 visual patient-reported outcomes and safety: camizestrant for emerging *ESR1*m advanced breast cancer during first-line endocrine-based therapy

**DOI:** 10.1093/oncolo/oyag278

**Published:** 2026-07-24

**Authors:** Adam Brufsky, Yeon Hee Park, François-Clément Bidard, Erica L Mayer, Wolfgang Janni, Cynthia Ma, Massimo Cristofanilli, Giampaolo Bianchini, Hiroji Iwata, Peter A Fasching, Zbigniew Nowecki, Javier Pascual, Shin-Cheh Chen, Lionel Moreau, Manuel Ruiz-Borrego, Ayelet Shai, Nuri Karadurmus, Kyung Hae Jung, Yuichiro Kikawa, Richard D Baird, Ranya Habash, Rebecca Sugarman, Steven Fox, Manuel Selvi Miralles, Cynthia Huang Bartlett, Nicholas Turner

**Affiliations:** Department of Medicine, UPMC Magee-Womens Hospital, Pittsburgh, PA 15213, United States; Samsung Medical Center, Sungkyunkwan University School of Medicine, Seoul 06351, Republic of Korea; Department of Medical Oncology, Institut Curie, 75005 Paris, France; Medical Oncology, Dana-Farber Cancer Institute, Boston, MA 02215, United States; Department of Gynecology and Obstetrics, Universitätsklinikum Ulm, 89081 Ulm, Germany; Department of Medicine, Washington University School of Medicine, St. Louis, MO 63110, United States; Medical Oncology, Weill Cornell Medicine/New York-Presbyterian Hospital, New York, NY 10065, United States; Department of Medical Oncology, IRCCS Ospedale San Raffaele, 20132 Milan, Italy; Department of Clinical Research Strategy, Nagoya City University, Nagoya 467-8601, Japan; Comprehensive Cancer Center Erlangen-EMN, University Hospital Erlangen, 91054 Erlangen, Germany; Department of Breast Cancer and Reconstructive Surgery, Narodowy Instytut Onkologii im. Marii Skłodowskiej-Curie, 02-781 Warsaw, Poland; Medical Oncology Department, Hospital Universitario Virgen de la Victoria, 29010 Málaga, Spain; Department of General Surgery, Chang Gung Medical Foundation Linkou Branch, 333 Taoyuan City, Taiwan; Medical Oncology, Pôle Santé République, 63100 Clermont-Ferrand, France; Oncology Department, Hospital Universitario Virgen del Rocio, 41013 Seville, Spain; Department of Oncology, Rambam Health Care Campus, Haifa 3109601, Israel; Medical Oncology Department, Gülhane Training and Research Hospital, University of Health Sciences, 06010 Ankara, Turkey; Asan Medical Center, University of Ulsan College of Medicine, Seoul 05505, Republic of Korea; Department of Breast Surgery, Kansai Medical University Hospital, Hirakata City, Osaka 573-1191, Japan; Department of Oncology, Cancer Research UK Cambridge Centre, Cambridge, CB2 0RE, United Kingdom; Department of Ophthalmology, Bascom Palmer Eye Institute/University of Miami, Miami, FL 33136, United States; Evinova, Oncology R&D, AstraZeneca, Waltham, MA 02451, United States; Late Development, Oncology R&D, AstraZeneca, Cambridge, CB2 0AA, United Kingdom; Late Development, Oncology R&D, AstraZeneca, Cambridge, CB2 0AA, United Kingdom; Late Development, Oncology R&D, AstraZeneca, Gaithersburg, MD 20878, United States; Breast Unit, Royal Marsden Hospital, London, SW3 6JJ, United Kingdom

**Keywords:** ophthalmology, vision tests, quality of life, patient reported outcome measure, breast neoplasms

## Abstract

**Background:**

We report ophthalmological assessments, patient-reported visual symptoms/functioning, and characterization of visual effect AEs from the SERENA-6 study.

**Materials and methods:**

This double-blind, placebo-controlled phase III study included 315 patients with ER-positive advanced breast cancer receiving first-line aromatase inhibitor (AI)+CDK4/6 inhibitor (CDK4/6i). Patients with emerging *ESR1* mutations in ctDNA and no radiological progression were randomized 1:1 to switch to camizestrant+CDK4/6i or continue AI+CDK4/6i. Predefined group term visual effect AEs were graded (NCI-CTCAE v5.0). Ophthalmological assessments were conducted at baseline, as indicated, and at the end of treatment. Analyses of patient-reported visual effects/functioning were exploratory.

**Results:**

Visual effect AEs were reported in 49 (31.6%) patients receiving camizestrant+CDK4/6i and 25 (16.1%) patients receiving AI+CDK4/6i; 90% were grade 1; none led to discontinuation. No changes in visual acuity or ocular structure were observed. Patient-reported visual effects occurred early (by week 2) and were reversible post-treatment. The proportion of patients in the camizestrant+CDK4/6i and AI+CDK4/6i arm reporting short-lived visual effects (<1 minute) ranged from 60% to 67% vs 50% to 69%, respectively; visual effects causing no/a low degree of bother: 78%-88% vs 50%-75%, respectively. During treatment, visual functioning was comparable to baseline and between arms.

**Conclusion:**

If experienced, patient-reported visual effects (including photopsia) with camizestrant+CDK4/6i were short-lived and reversible post-treatment. Visual effect AEs with camizestrant+CDK4/6i were mostly mild and did not require treatment discontinuation or ophthalmological management. There was no impact on ocular structure, function, or visual acuity; visual effects had little/no impact on daily functioning. These findings support clinical decision-making by further characterizing the visual safety profile of camizestrant in this setting.

**Clinical trial registration:**

ClinicalTrials.gov, NCT04964934.

## Graphical Abstract

**Graphical Abstract:**
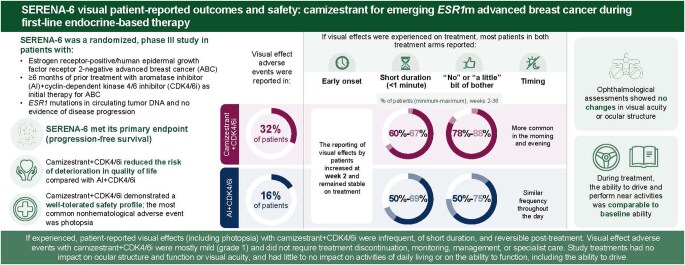

Implications for Practice This detailed analysis of visual effect adverse events and comprehensive ophthalmological assessments from the SERENA-6 trial demonstrated that there was no change in ocular structure or visual acuity with camizestrant+CDK4/6i treatment. Patient-reported outcome questionnaires demonstrated that visual effects caused little to no impact on daily activity and functioning. Collectively, these data support a symptom-guided approach rather than routine ophthalmological examination. Patients should be educated about photopsia and reporting new visual symptoms, and reminded of the importance of general overall eyecare, regardless of treatment, for any progressive, persistent, or unilateral symptoms.

## Introduction

Camizestrant is a next-generation oral selective estrogen receptor degrader and complete estrogen receptor antagonist that was designed to have activity against *ESR1* mutations (*ESR1*m) as well as wild-type estrogen receptor.^1^^,^^2^ The phase III, randomized, double-blind SERENA-6 trial was the first global registrational breast cancer study to use circulating tumor DNA (ctDNA) monitoring to identify the emergence of an acquired resistance mutation—in this case *ESR1*m—before progression and direct a change in therapy.^3^ At an interim analysis, switching to camizestrant from aromatase inhibitor (AI) with continued cyclin-dependent kinase 4/6 inhibitor (CDK4/6i) at *ESR1*m emergence during first-line therapy, ahead of disease progression, resulted in a statistically significant and clinically meaningful improvement in progression-free survival compared with continuation of AI+CDK4/6i (median: 16.0 months vs 9.2 months; hazard ratio: 0.44 [95% confidence interval (CI), 0.31-0.60; stratified log-rank test *P* < .0001]) (data cutoff: November 28, 2024).^3^ Progression-free survival results were consistent with longer follow-up (median: 16.8 months vs 9.2 months; hazard ratio: 0.45 [95% CI, 0.34-0.59; nominal *P *< .00001]) (data cutoff: January 2, 2026).^4^ Camizestrant+CDK4/6i also delayed time to deterioration in cancer symptoms (pain, fatigue, dyspnea), functioning (physical, role, and emotional), and global health status and quality of life (hazard ratio: 0.54 [95% CI, 0.34-0.84]).^5^

Visual effect adverse events (AEs) with camizestrant 75 mg monotherapy have been reported in some early phase trials in patients with estrogen receptor-positive/human epidermal growth factor receptor 2 (HER2)-negative advanced breast cancer,^6^^,^^7^ the most common being photopsia^6^ (very brief flashes of light in the periphery of vision). Photopsia was also the most common nonhematological AE following treatment with camizestrant+CDK4/6i in SERENA-6^3^; when reported, events were mostly grade 1 (90%). If experienced, photopsia AEs in SERENA-6 were well tolerated, as no patients discontinued treatment due to these events.^3^

Here, we report a detailed analysis of visual effects from the SERENA-6 trial, including timing and grade, as well as results from various ophthalmological assessments (data cutoff: November 28, 2024). We also report a detailed analysis of results from two patient-reported outcome questionnaires that assessed visual effects, including the impact on activities of daily living and visual function.

## Materials and methods

### Study design and patients

The SERENA-6 study design and primary efficacy and safety results have been previously reported.^3^ Briefly, SERENA-6 is a phase III, global, randomized, double-blind, placebo-controlled study that assessed the safety and efficacy of switching from AI to camizestrant with continuation of CDK4/6i during first-line therapy at emergence of *ESR1*m and ahead of disease progression. Adult patients with estrogen receptor-positive/HER2-negative advanced breast cancer who had received ≥6 months of first-line AI+CDK4/6i for metastatic disease were tested for *ESR1*m in ctDNA every 2-3 months (Guardant360^®^ CDx, Guardant Health, Palo Alto, CA, USA), coinciding with routine clinical assessments. Patients with *ESR1*m and no evidence of radiological or clinical disease progression and who met other eligibility criteria entered the double-blind randomized treatment period.

The study was conducted in accordance with the Declaration of Helsinki and the applicable International Council for Harmonisation of Technical Requirements for Pharmaceuticals for Human Use and Good Clinical Practice guidelines. All patients gave informed consent before enrollment. The study protocol, amendments, and other relevant documents were reviewed by an institutional review board and an independent ethics committee.

### Study treatment

Eligible patients were randomized 1:1 to continue to receive AI+CDK4/6i with matching camizestrant placebo or to switch to camizestrant (75 mg once daily) + CDK4/6i (same type and dose) with matching AI placebo. Premenopausal or perimenopausal women, and men, continued to receive a luteinizing hormone-releasing hormone agonist.

One dose reduction of camizestrant/placebo from 75 mg to 50 mg was allowed per patient. Dose reductions of CDK4/6i were in line with the local prescribing label and were not permitted for AI. Per protocol, grade 1 visual effects did not require dose modification for camizestrant/placebo. For grade ≥2 visual effects, the protocol mandated a dose interruption of camizestrant/placebo until resolution of symptoms and, if deemed appropriate, referral for ophthalmological assessment. Camizestrant/placebo could then be restarted as medically indicated at the same or a reduced dose, and visual effects reassessed at the next visit.

### Safety

Treatment-emergent AEs were defined as AEs with an onset or that worsen (per investigator opinion) from the first dose of trial treatment until 4 weeks post-discontinuation, and prior to the start of any subsequent cancer therapy. AEs were recorded continuously until 4 weeks post-discontinuation of a trial treatment and were graded according to the National Cancer Institute Common Terminology Criteria for Adverse Events version 5.0. The visual effect group term comprised the preferred terms photopsia, vision blurred, visual impairment, diplopia, photophobia, visual perseveration, and glare.

### Ophthalmological assessments

Ophthalmological assessments were conducted at baseline (where feasible) or until day 1 of cycle 2, as clinically indicated during treatment, and at treatment discontinuation. Assessments included best-corrected (manifest refraction) and uncorrected near and distance visual acuities in left and right eyes. Visual acuity was assessed in lines on standardized eye charts. Consistent with US Food and Drug Administration and National Eye Institute/Early Treatment Diabetic Retinopathy Study practice, a loss of ≥3 lines from baseline in best-corrected distance visual acuity was considered a clinically meaningful deterioration in vision. Ophthalmological assessments also included: pupillary light reflex, slit lamp, intraocular pressure measurements, dilated fundoscopy exam, and optical coherence tomography. Further information on additional ophthalmological assessments is provided in the Supplementary Methods. An independent advisory committee of ocular experts was consulted regularly and evaluated visual effect AEs and specific ocular data.

### Patient-reported outcome measures and assessment

Evaluation of vision-related symptoms and functioning as measured by the Visual Symptom Assessment Questionnaire (VSAQ) and the National Eye Institute Visual Function Questionnaire-25 (NEI-VFQ-25) were predefined exploratory endpoints. Patients completed patient-reported outcome questionnaires electronically at home or at the clinic if the time of assessment coincided with a scheduled visit; proxy-reported patient outcomes were not allowed. An advisory committee of ocular experts was involved in the selection of visual patient-reported outcome questionnaires.

The VSAQ is a 14-item questionnaire that assesses the presence, frequency, timing, duration, and degree of bother of visual effects and their impact on activities of daily living.^8^ The VSAQ was selected as the most appropriate patient-reported outcome tool to measure these aspects of visual effects based on ophthalmological assessments and patient interviews that were performed in SERENA-1 and SERENA-2. Patients completed the VSAQ on day 1 of week 1, week 2, week 6, week 24, and every 12 weeks thereafter until treatment discontinuation, at the treatment discontinuation visit, and 4 weeks post-treatment discontinuation. Only patients who responded “Yes” to experiencing a visual effect in the past week answered the subsequent questions. The VSAQ had a recall period of 1 week. The impact of visual effects on activities of daily living was measured using a 10-point scale (0: no effect; 10: completely prevented activity of daily living).

The NEI-VFQ-25 is a validated 25-item questionnaire that was chosen to assess vision-related health status.^9^ The NEI-VFQ-25 comprises 11 subscales that assess global vision, the impact of vision on different aspects of functioning, peripheral vision, color vision, ocular pain, and general health. Patients completed the NEI-VFQ-25 at baseline (day 1 of week 1), every 6 months thereafter until treatment discontinuation, and at the treatment discontinuation visit.

The VSAQ was administered more frequently because it was intended to capture treatment-emergent or changing visual symptoms, including early changes after treatment initiation and changes around treatment discontinuation. In contrast, the NEI-VFQ-25 was administered at longer intervals because it assesses broader vision-related functioning and quality of life, which are expected to change more gradually. Less frequent assessment, when not necessary, also reduces patient burden.

### Statistical analysis

The safety population included all patients who had received ≥1 dose of camizestrant, CDK4/6i, AI, or placebo; this population was also used for patient-reported outcome analyses. Safety data, including those from ophthalmological assessments, were descriptively summarized.

Visual patient-reported endpoints were exploratory and were not prospectively powered for statistical comparison or controlled for type I error; therefore, no formal claims of statistical significance can be made for these endpoints. Adherence to visual patient-reported outcome questionnaires was calculated for each time point, defined as the number of patients with an evaluable questionnaire (≥1 subscale completed) divided by the total number of patients expected to complete the questionnaire at that time point. The VSAQ responses were summarized descriptively at all time points at which ≥10 patients in either arm had experienced a visual disturbance in the last week and went on to complete the remainder of the questionnaire. The score and change from baseline of the impact on activities of daily living were also summarized descriptively. Scores for each item on the NEI-VFQ-25 were converted to a 0-100 scale, with a higher score representing better functioning.^9^ Items within each subscale were averaged to create the subscale scores. The composite score was calculated by averaging the 11 vision-targeted subscales, excluding the general health score.

## Results

### Patient characteristics

The SERENA-6 study population included 315 patients with emergent *ESR1*m during first-line therapy and no evidence of disease progression (Figure S1). Patients were screened for inclusion in the trial between June 2021 and June 2024 and randomized to switch to camizestrant+CDK4/6i (157 patients) or to continue receiving AI+CDK4/6i (158 patients). Median duration of follow-up at data cutoff was 12.6 months.^3^ The characteristics of the overall study population have been previously published.^3^

The safety population included 310 patients who had received any trial treatment (155 patients in each treatment arm). Two patients in the camizestrant+CDK4/6i arm and three patients in the AI+CDK4/6i arm were randomized but did not subsequently receive treatment. Among randomized patients, the median age was 61 years (range: 29-89), and 26/157 (16.6%) patients in the camizestrant+CDK4/6i arm and 26/158 (16.5%) patients in the AI+CDK4/6i arm had an ongoing eye disorder at baseline.

### Visual effects by AE reporting

Visual effect AEs were reported in 49 (31.6%) patients treated with camizestrant+CDK4/6i and 25 (16.1%) patients treated with AI+CDK4/6i (Table 1). The majority of visual effect AEs were grade 1 in both the camizestrant+CDK4/6i (*n *= 44/49) and the AI+CDK4/6i arms (*n *= 24/25). A grade 3 visual effect AE was reported in only one patient in the camizestrant+CDK4/6i arm (photopsia, limiting instrumental activities of daily living). This event occurred at night in artificial light and resolved. There were no grade 4 visual effect AEs reported in either treatment arm.

**Table 1.** Summary of all visual effect AEs.

AE, *n* (%)	Camizestrant+CDK4/6i (*n *= 155)				AI+CDK4/6i (*n *= 155)			
	Any grade	Grade 1	Grade 2	Grade 3	Any grade	Grade 1	Grade 2	Grade 3
**Any**	49 (31.6)	44 (28.4)	4 (2.6)	1 (0.6)	25 (16.1)	24 (15.5)	1 (0.6)	0
**Photopsia**	31 (20.0)	28 (18.1)	2 (1.3)	1 (0.6)	12 (7.7)	11 (7.1)	1 (0.6)	0
**Vision blurred**	11 (7.1)	9 (5.8)	2 (1.3)	0	11 (7.1)	11 (7.1)	0	0
**Visual impairment**	6 (3.9)	6 (3.9)	0	0	3 (1.9)	3 (1.9)	0	0
**Diplopia**	3 (1.9)	3 (1.9)	0	0	2 (1.3)	2 (1.3)	0	0
**Photophobia**	3 (1.9)	3 (1.9)	0	0	0	0	0	0
**Visual perseveration**	2 (1.3)	2 (1.3)	0	0	0	0	0	0

AEs regardless of causality. Glare was not reported in patients from either arm. There were no grade 4 visual effect AEs in either arm.

Abbreviations: AE, adverse event; AI, aromatase inhibitor; CDK4/6i, cyclin-dependent kinase 4/6 inhibitor.

The most commonly reported visual effect AE (preferred term) in either arm was photopsia (20% with camizestrant+CDK4/6i and 7.7% with AI+CDK4/6i), and ≥90% of these AEs were grade 1 (Table 1).

Rates of other AEs in the visual effect group term are reported in Table 1. All other AEs in the visual effect group term were grade 1, except in two patients receiving camizestrant+CDK4/6i who reported grade 2 blurred vision (Table 1). Serious visual effect AEs were reported in no patients in the camizestrant+CDK4/6i arm and in one patient in the AI+CDK4/6i arm (visual impairment, grade 1).

The median number of visual effect event AEs was 1 (interquartile range [IQR]: 1-2) in the camizestrant+CDK4/6i arm and 1 (IQR: 1-1) in the AI+CDK4/6i arm. The median time to onset of a visual effect AE was 8.0 days (IQR: 3-16) with camizestrant+CDK4/6i and 23.0 days (IQR: 14-100) with AI+CDK4/6i.

Visual effect AEs led to a dose interruption in four (2.6%) patients receiving camizestrant+CDK4/6i and one (0.6%) patient receiving AI+CDK4/6i and did not lead to dose reductions or treatment discontinuation in either treatment arm. Visual effect AEs did not require monitoring, management with additional treatment, or specialist involvement.

### Ophthalmological assessments

#### Visual acuity

Data for best-corrected distance visual acuity at baseline and ≥1 post-baseline assessment were available for 35 and 36 patients in the camizestrant+CDK4/6i arm for the left and right eyes, respectively, and 35 patients in the AI+CDK4/6i arm for both eyes. In either arm, the majority of patients with a baseline and ≥1 on-treatment assessment had a < 1-line loss from baseline in best-corrected distance visual acuity, which was considered no clinically meaningful change from baseline in visual acuity (Table 2). Clinically significant changes (≥3-line loss) per investigator opinion in visual acuity were rare (≤6%) and balanced between treatment arms, suggesting no drug-related effect on visual acuity with camizestrant+CDK4/6i. No patient experienced bilateral or progressive ≥3-line loss in best-corrected distance visual acuity in either eye.

**Table 2.** Summary of ophthalmological assessments.

	Camizestrant+CDK4/6i		AI+CDK4/6i	
	Left eye	Right eye	Left eye	Right eye
**Worst change from baseline in best-corrected visual acuity, *n* (%)**				
**Patients with a baseline assessment and ≥1 post-baseline assessment, *n***	35	36	35	35
**Patients with no change or a decrease in visual acuity, *n***	32	31	29	30
** <1-line loss^a^**	27 (84.4)	26 (83.9)	22 (75.9)	23 (76.7)
** 1-line loss**	3 (9.4)	2 (6.5)	5 (17.2)	5 (16.7)
** 2-line loss**	0	2 (6.5)	1 (3.4)	1 (3.3)
** ≥3-line loss**	2 (6.3)	1 (3.2)	1 (3.4)	1 (3.3)
**Change between baseline and worst on-treatment assessment, *n* (%)**				
**Fundoscopy, *n***	39		38	
** No clinically meaningful change**	36 (92.3)		38 (100)	
** Improvement**	2 (5.1)		0	
** Worsening**	1 (2.6)		0	
**Slit lamp examination, *n***	36		35	
** No clinically meaningful change**	31 (86.1)		33 (94.3)	
** Improvement**	0		1 (2.9)	
** Worsening**	5 (13.9)		1 (2.9)	
**Pupillary light reflex test, *n***	36		32	
** No clinically meaningful change**	36 (100)	36 (100)	32 (100)	32 (100)
** Improvement**	0	0	0	0
** Worsening**	0	0	0	0
**Extraocular muscle movement test, *n***	37		32	
** No clinically meaningful change**	37 (100)	37 (100)	32 (100)	32 (100)
** Improvement**	0	0	0	0
** Worsening**	0	0	0	0
**Optical coherence tomography, *n***	31		33	
** No clinically meaningful change**	30 (96.8)	29 (93.5)	33 (100)	33 (100)
** Improvement**	1 (3.2)	2 (6.5)	0	0
** Worsening**	0	0	0	0
**Mean (SD) intraocular pressure, mmHg**				
** Baseline**	*n *= 92		*n *= 92	
	16.8 (19.6)	16.7 (17.3)	14.8 (3.4)	15.0 (3.2)
** End of treatment^b^**	*n *= 56		*n *= 72	
	19.9 (37.6)	18.8 (33.7)	14.8 (2.7)	14.6 (3.0)
** Change from baseline**	*n *= 30		*n *= 32	
	−0.5 (2.4)	−1.9 (6.2)	−0.5 (3.1)	−0.7 (2.9)

Worst change from baseline in best-corrected distance visual acuity: data are shown only for patients with a paired baseline and post-baseline assessment. Percentages are calculated from patients who had no change or a decrease in visual acuity.

Abbreviations: AI, aromatase inhibitor; CDK4/6i, cyclin-dependent kinase 4/6 inhibitor; SD, standard deviation.

a A <1-line loss was considered no clinically meaningful change from baseline in visual acuity. Change between baseline and worst on-treatment assessment: categories are based on worst on-treatment values comparable to baseline assessments per investigator assessment. “No clinically meaningful change” includes on-treatment values comparable to baseline assessments. “Improvement” includes abnormal clinically significant baseline values normalizing or becoming nonclinically significant on treatment. “Worsening” includes normal or abnormal nonclinically significant baseline values becoming abnormal and clinically significant on treatment. An intraocular pressure increase to ≥25 mmHg would be considered clinically meaningful.

b The end-of-treatment value is the last non-missing assessment between the start of treatment and 35 days following the last dose of study treatment.

Consistent with investigator findings, an independent ophthalmological safety review reported that camizestrant+CDK4/6i was not associated with changes in visual acuity. Visual acuity remained stable following camizestrant+CDK4/6i treatment, with clinically meaningful losses (≥3 lines) occurring in 4.1%-6.0% of eyes, which was balanced across treatment arms.

#### Other ophthalmological assessments

The majority of patients had no clinically meaningful change from baseline when assessed using dilated fundoscopy, slit lamp, pupillary testing, extraocular muscle movements, and optical coherence tomography (Table 2). Clinically significant findings (per investigator opinion) were generally rare, unilateral, nonprogressive, and occurred at similar frequencies in either treatment arm. No patients had drug-related visual field defects. There were no increases in intraocular pressure from baseline in either arm (Table 2). No ophthalmological abnormalities were reported as a serious AE, and no ocular events led to treatment discontinuation.

Review of the data by an independent expert ophthalmologist reported that across ophthalmological modalities, camizestrant+CDK4/6i was not associated with ocular structural changes. Post-baseline findings were predominantly normal or not clinically significant. In patients with a visual effect AE, ophthalmological assessments near symptomatic periods (within 7 days) were normal or not clinically significant.

### Patient-reported outcomes

#### Adherence

At baseline, 68% of patients in the camizestrant+CDK4/6i arm and 60% of patients in the AI+CDK4/6i arm completed ≥1 subscale of the VSAQ. Adherence to the VSAQ increased post-baseline and remained consistent and similar between treatment arms. The range of adherence rates post-baseline up to week 36 was 73%-81% for the camizestrant+CDK4/6i arm and 70%-74% for the AI+CDK4/6i arm (Figure S2A).

At baseline, 69% of patients in the camizestrant+CDK4/6i arm and 59% of patients in the AI+CDK4/6i arm completed ≥1 subscale of the NEI-VFQ-25 (Figure S2B). Adherence to the NEI-VFQ-25 remained consistent post-baseline and was similar between treatment arms.

#### Patterns and timing of patient-reported visual effects

**Figure 1.**
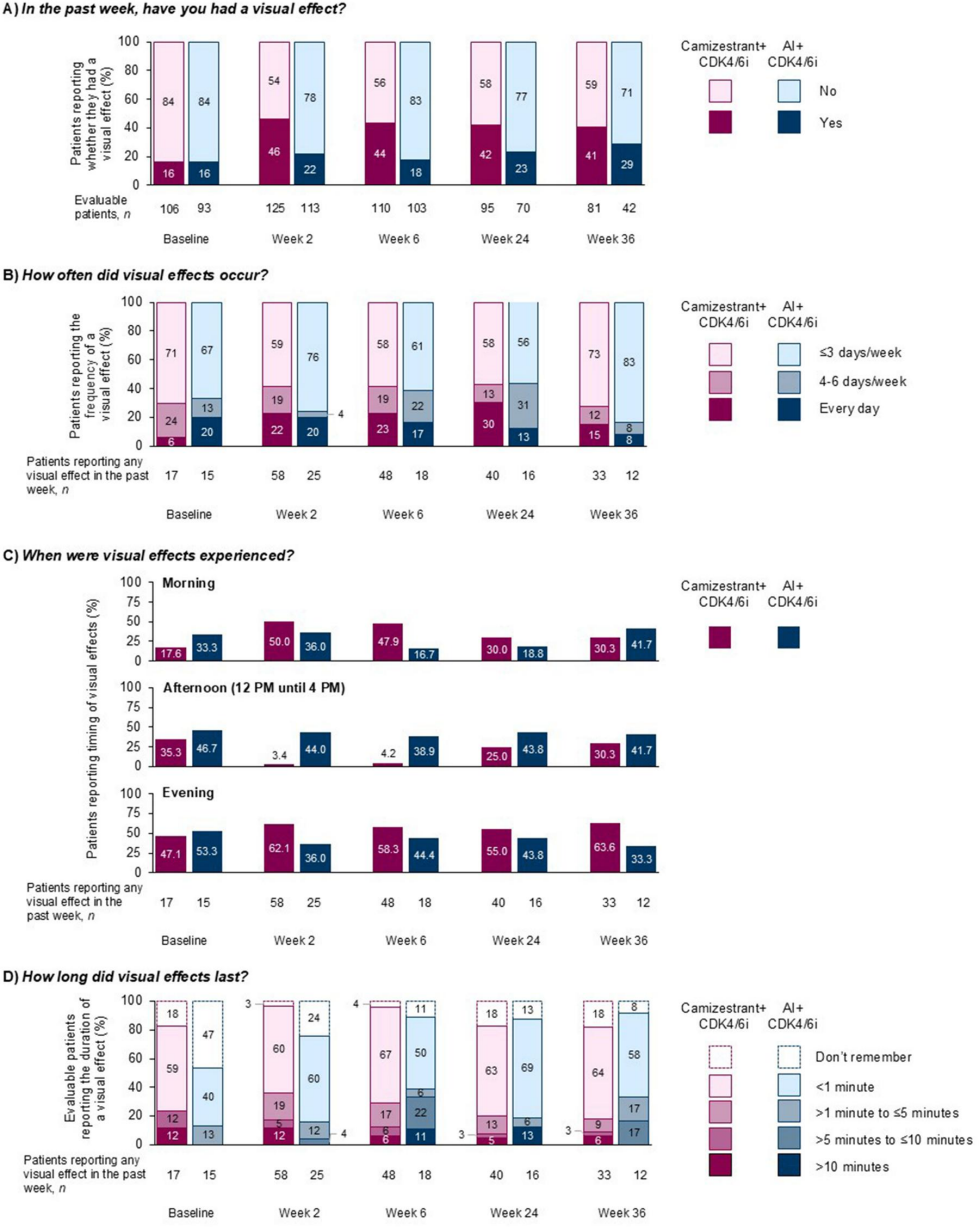
Incidence (A), frequency (B), timing (C), and duration (D) of patient-reported visual effects, as measured by the VSAQ, by treatment visit. Data are shown from time points where there were data from ≥10 patients in either arm. Only patients reporting any visual effect in the past week and who completed the questionnaire items are shown in (B-D). Some totals may not equal 100% due to rounding. For (C), patients could have chosen >1 response. For (D), responses of “<30 seconds” and “>30 seconds but <1 minute” were combined into the category “<1 minute.” Abbreviations: AI, aromatase inhibitor; CDK4/6i, cyclin-dependent kinase 4/6 inhibitor; VSAQ, Visual Symptom Assessment Questionnaire.

At baseline, 16% of patients in each treatment arm reported a visual effect in the past week in the VSAQ. Of patients who experienced a visual effect in the camizestrant+CDK4/6i arm, the rate of reporting was highest at week 2 (46%), after which the proportion of patients reporting visual effects remained stable until 4 weeks post-discontinuation of camizestrant+CDK4/6i, where levels reduced to below baseline levels (7%) (Figure 1A). The pattern of patient-reported visual effects in the AI+CDK4/6i arm was stable over time. Of patients who experienced a visual effect with camizestrant+CDK4/6i, the majority reported that they occurred ≤3 days per week (range [min-max] week 2-36: 58%-73%; Figure 1B), in the morning or evening (morning range: 30%-50%; evening range: 55%-64%; Figure 1C), and lasted <1 minute per episode (60%-67%; Figure 1D). Similar results were observed in the AI+CDK4/6i arm, with the exception that visual effects occurred at a similar frequency during the day (Figure 1C).

#### Patient-reported visual effect symptoms and visual status

In either arm, >80% of patients who experienced a visual effect reported that they “never/rarely” or “sometimes” had a visual effect when adjusting to changes in lighting across all time points (range [min-max] week 2-36: camizestrant+CDK4/6i, 83%-88%; AI+CDK4/6i, 92%-96%) (Figure S3A). Across all time points, the majority of patients in either arm who experienced a visual effect reported that they were “not at all” or “a little” bit bothered by overlapping shadows, shimmering, flashes of light, streamers, strings or floaters, difficulty seeing at night, hazy or blurry vision, or difficulty adapting to bright or dim light (Figure S3B-H).

**Figure 2.**
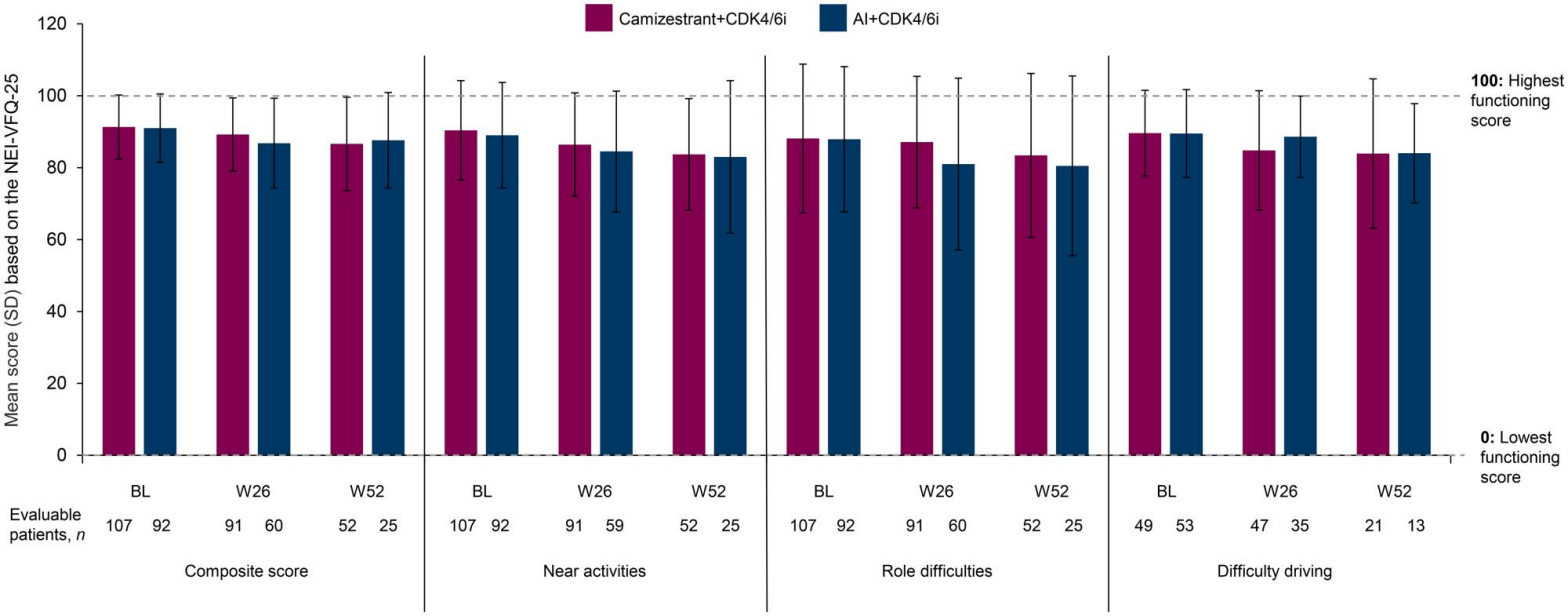
Responses to select subscales of the NEI-VFQ-25, by treatment visit. The scores range from 0 to 100, with a higher score representing better functioning. The composite score is the average of the vision-targeted subscale scores, excluding the general health score. Abbreviations: AI, aromatase inhibitor; BL, baseline; CDK4/6i, cyclin-dependent kinase 4/6 inhibitor; NEI-VFQ-25, National Eye Institute Visual Function Questionnaire-25; SD, standard deviation; W, week.

Patient-reported vision-related health status, as measured by the NEI-VFQ-25 composite score, was stable over time. Mean composite scores remained ≥87 across all time points and were comparable between treatment arms (Figure 2). Peripheral vision, color vision, global vision rating, and ocular pain scores were similar to baseline scores and comparable between treatment arms (Figure S4).

#### Impact of patient-reported visual effects on daily life

**Figure 3.**
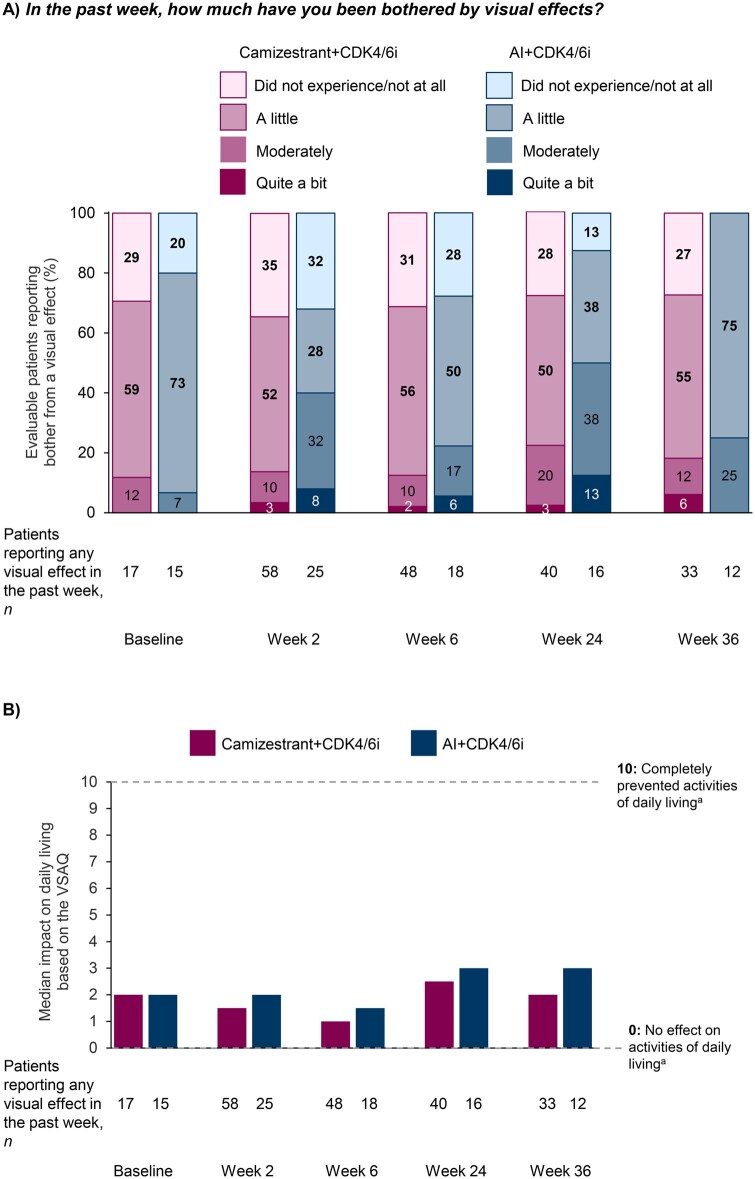
Degree of bother (A) and impact on daily living by patient-reported visual effects (B), as measured by the VSAQ, by treatment visit. Data are shown from time points where there were data from ≥10 patients in either arm. Only patients reporting any visual effect in the past week completed the questionnaire items shown in (A). In (A), no patients from either arm reported being extremely bothered by visual effects while on study treatment. In (B), activity of daily living score was measured using a 10-point scale (0: no effect; 10: completely prevented activity of daily living). Some totals may not equal 100% due to rounding. ^a^Example of activities of daily living given in the VSAQ include housework, shopping, childcare, exercising, or studying. Abbreviations: AI, aromatase inhibitor; CDK4/6i, cyclin-dependent kinase 4/6 inhibitor; VSAQ, Visual Symptom Assessment Questionnaire.

Across all on-treatment time points, the majority of patients who experienced a visual effect in either treatment arm reported that they were “not at all” or “a little” bit bothered by visual effects (range [min-max] week 2-36: camizestrant+CDK4/6i, 78%-88%; AI+CDK4/6i, 50%-75%) (Figure 3A). No patients were “extremely bothered” by visual effects in either arm.

Of patients who experienced a visual effect, the median impact of visual effects on activities of daily living was minimal in both arms, and scores were maintained across time points (range [min-max] week 2-36: camizestrant+CDK4/6i, 1.0-2.5; AI+CDK4/6i, 1.5-3.0) (Figure 3B).

Ability to drive, including in familiar places and in bad conditions, and perform near activities (eg, reading, sewing) and distant activities (eg, watching movies) was comparable to baseline and between treatment arms (Figure 2). Dependency, mental health, and social and role functioning scores were also similar to baseline scores and comparable between treatment arms (Figures 2 and S4).

## Discussion

SERENA-6 is the first phase III, double-blind, placebo-controlled study investigating camizestrant in combination with CDK4/6i. At the primary analysis, camizestrant+CDK4/6i demonstrated a well-tolerated safety profile. As photopsia was the most commonly reported nonhematological AE, we characterized visual effect AEs and reported a detailed analysis of ophthalmological assessments, as well as patient-reported outcomes of vision-related symptoms and functioning.

We found that the vast majority (90%) of visual effect AEs with camizestrant+CDK4/6i treatment were grade 1. Visual effect AEs with camizestrant+CDK4/6i in SERENA-6 were transient, reversible, and resulted in a very low rate of dose interruptions (2.6%) and no treatment discontinuations, in line with previous reports.^6^^,^^7^^,^^10^ Visual effect AEs were commonly reported in both treatment arms but more so in the camizestrant+CDK4/6i arm (32%) than the AI+CDK4/6i arm (16%). Onset of visual effect AEs occurred early during treatment with camizestrant+CDK4/6i (median onset: <2 weeks), consistent with previous reports^6^; the median time to onset was earlier than that observed in the AI+CDK4/6i arm (median onset: ∼3 weeks). No patients in SERENA-6 required additional monitoring for visual effect AEs or management with additional treatment, further supporting that visual effects with camizestrant+CDK4/6i are of low clinical significance. Patients should be educated about photopsia and reporting new visual symptoms, and reminded of the importance of general overall eyecare, regardless of treatment, for any progressive, persistent, or unilateral symptoms.

Results from the ophthalmological assessments, including temporally matched assessments, collectively demonstrated that there is no impact on ocular structure (including retina, optic nerve, and macular), function, or visual acuity with camizestrant+CDK4/6i treatment, consistent with previous reports.^3^^,^^6^^,^^7^^,^^10^ Clinically meaningful changes in unilateral visual acuity were rare, nonprogressive, and comparable to the AI+CDK4/6i arm. Almost all patients retained a normal/clinically insignificant value following treatment when assessed using a variety of modalities. Investigator-reported ophthalmological findings were consistent with those from an independent expert ophthalmologist review.

Nonclinical studies in rats demonstrated a reversible pharmacological effect of camizestrant on intracellular retinal responses during light transitions in the absence of structural changes.^11^ The available clinical data showing that visual effects with camizestrant (primarily reported as photopsia) are transient, reversible, and without accompanying structural changes^3^^,^^6^^,^^7^^,^^10^ align with the finding from nonclinical studies.^11^

The patient’s subjective experience of symptoms, AEs, and their ability to function is increasingly being evaluated alongside efficacy and safety results to support the benefit–risk profile of novel anticancer treatments. In SERENA-6, camizestrant+CDK4/6i prolonged the time to deterioration in global health status and quality of life compared with AI+CDK4/6i.^3^^,^^5^ Our results strengthen the body of patient-reported outcome data with this analysis from two visual-specific patient-reported outcome questionnaires.

In both treatment arms, the proportion of patients reporting an on-treatment visual effect with the VSAQ was higher than AE reporting of a visual effect. Discrepancies between clinician-reported and patient-reported AEs are well documented.^12–14^ Furthermore, visual effects as reported in the VSAQ were not defined and could include visual effects that were not part of the visual effect AE group term, which may account for the higher proportion of patients reporting a visual effect compared with AE reporting.

Consistent with the safety data, patients reported that visual effects, if experienced, occurred early, by week 2 of treatment. Patients reported that visual effects with camizestrant+CDK4/6i had little impact on their quality of life and functioning, as evidenced by the majority of patients reporting that these visual effects occurred infrequently, for short periods, and were associated with little to no bother, as assessed by the VSAQ. A notable difference between arms was that patient-reported visual effects occurred at a similar frequency throughout the day in the AI+CDK4/6i arm, whereas they were predominantly experienced in the morning and evening in the camizestrant+CDK4/6i arm. Nonclinical studies in rats reported that camizestrant was associated with functional changes in retinal responses during light transitions, which may support the finding that patient-reported visual effects were more common in the morning and evening, when light conditions are changing.^11^ Consistent with the patient experience as captured by the VSAQ, responses from the NEI-VFQ-25 show that visual effects with camizestrant+CDK4/6i had no impact on driving, near activities, or role function, and that the ability to perform these functions was stable across treatment and comparable with patients receiving AI+CDK4/6i.

The double-blind, placebo-controlled study design of SERENA-6 is the gold standard and allowed for unbiased assessment of AEs following treatment and subjective AEs in both arms. While photopsia has been considered a camizestrant-associated AE,^6^^,^^7^ visual effects were reported in 16% of patients in the AI+CDK4/6i arm; the placebo-controlled study design makes it unlikely that functional unblinding of the patient/clinician would lead to altered expectation biases, amplified treatment effects, or affect the integrity of clinical assessments. Visual effect AEs, such as vision blurred, following treatment with CDK4/6i has been documented^15–18^ and may contribute to the incidence of visual effect AEs in both arms. The study prospectively embedded comprehensive, multimodal ophthalmological safety assessments into a large, randomized, controlled study so the visual signal was characterized in a robust comparative framework. Results from ophthalmological analyses were reported in patients with paired baseline and on-treatment samples, allowing within-subject ocular changes following treatment to be determined. While missing data is a potential limitation, on-treatment ophthalmological analyses were not compulsory, and therefore paired samples from all patients were not expected. The number of patients with paired samples was balanced between arms. Of note, an independent expert ophthalmologist review confirmed that the available paired structural assessments, together with temporally matched assessments around symptomatic events, were adequate for the detection of an ophthalmological safety signal. An additional strength of this study is that patients with prior ophthalmological conditions were included in the analysis, which means results reported here can be generalized to a broader patient population. Other strengths of this study include the use of two visual-specific patient-reported outcome measures that provide a comprehensive picture of patient visual-related symptoms and their impact on quality of life. The characteristics of patients in this study were also considered largely representative of patients with hormone receptor (HR)-positive/HER2-negative breast cancer.^19–21^ A limitation of this study is that assessment of patient-reported visual effects and visual functioning with the VSAQ and the NEI-VFQ-25 were exploratory endpoints. A further limitation is the potential for selection bias introduced by missing data to influence patient-reported outcome findings. However, questionnaire adherence rates were similar between treatment arms (∼65%) and remained relatively consistent throughout treatment, which suggests that missing data are unlikely to bias the findings reported here.

## Conclusion

In SERENA-6, visual effects were reported in both arms of the study. Despite being more common in patients receiving camizestrant+CDK4/6i than in patients receiving AI+CDK4/6i, detailed analysis demonstrates that visual effects (including photopsia) following treatment with camizestrant+CDK4/6i occurred early during treatment, were grade 1 in 90% of instances, did not require treatment discontinuations, showed no structural effect during comprehensive ophthalmological assessments, and had little to no impact on activities of daily living or ability to function, including the ability to drive. Together with the clinical efficacy and well-tolerated safety profile of camizestrant, these results further support the positive benefit–risk profile of switching to camizestrant while continuing CDK4/6i at emergence of *ESR1*m during first-line AI+CDK4/6i and ahead of disease progression in patients with HR-positive/HER2-negative advanced breast cancer.

## Supplementary Material

oyag278_Supplementary_Data

## Data Availability

Data underlying the findings described in this manuscript may be obtained in accordance with AstraZeneca’s data sharing policy described at https://www.astrazenecaclinicaltrials.com/our-transparency-commitments/. Data for studies directly listed on Vivli can be requested through Vivli at www.vivli.org. Data for studies not listed on Vivli could be requested through Vivli at https://vivli.org/members/enquiries-about-studies-not-listed-on-the-vivliplatform/. AstraZeneca Vivli member page is also available outlining further details: https://vivli.org/ourmember/astrazeneca/.
