# Supplementary material for: SERENA-6 visual patient-reported outcomes and safety: camizestrant for emerging ESR1m advanced breast cancer during first-line endocrine-based therapy
Source: Oncologist. 2026 Jul 24;31(9):oyag278. doi: 10.1093/oncolo/oyag278 (PMC13471996; doi:10.1093/oncolo/oyag278)
Supplement: Supplementary file 1 [file oyag278_supplementary_data.docx]

**SUPPLEMENTARY APPENDIX**

This appendix has been provided by the authors to give readers additional information about their work.

**Supplement to:**

**SERENA-6 visual patient-reported outcomes & safety: camizestrant for emerging ESR1m advanced breast cancer during first-line endocrine-based therapy**

Adam Brufsky, Yeon Hee Park, François-Clément Bidard, Erica L. Mayer, Wolfgang Janni, Cynthia Ma, Massimo Cristofanilli, Giampaolo Bianchini, Hiroji Iwata, Peter A. Fasching, Zbigniew Nowecki, Javier Pascual, Shin-Cheh Chen, Lionel Moreau, Manuel Ruiz-Borrego, Ayelet Shai, Nuri Karadurmus, Kyung Hae Jung, Yuichiro Kikawa, Richard Baird, Ranya Habash, Rebecca Sugarman, Steven Fox, Manuel Selvi Miralles, Cynthia Huang Bartlett, Nicholas Turner

*Correspondence to:*

Adam Brufsky, UPMC Magee-Womens Hospital, 300 Halket Street Pittsburgh, PA, 15213, USA
Tel: 412-641-4530
E-mail: brufskyam@upmc.edu

**CONTENTS**

[**SUPPLEMENTARY APPENDIX**	1](#_Toc234923127)

[**SUPPLEMENTAL METHODS**	3](#_Toc234923128)

[**SUPPLEMENTAL FIGURES**	4](#_Toc234923129)

[**Figure S1.** CONSORT diagram.	4](#_Toc234923130)

[**Figure S2.** Adherence rates by treatment visit for VSAQ (A) and NEI-VFQ-25 (B).	5](#_Toc234923131)

[**Figure S3.** Incidence of patient-reported visual effects with lighting changes and degrees of bother by specific visual symptoms, as measured by the VSAQ, by treatment visit.	6](#_Toc234923132)

[**Figure S4.** Responses to additional NEI-VFQ-25 items, by treatment visit.	10](#_Toc234923133)

**SUPPLEMENTAL METHODS**

**Ophthalmologic assessments**

Ophthalmological assessments included: current glasses/lens prescription (if applicable); extraocular muscle movements; confrontation visual fields; full anterior segment examinations with a slit lamp, including fluorescein or comparable vital staining of the cornea, with evaluation of orbit/lids/adnexa, conjunctiva, cornea, anterior chamber, iris, lens, and vitreous body; dilated fundoscopy exam, including the optic nerve, retina, vessels, and periphery, unless medically contraindicated; and evaluation of dry eye per local standard using slit lamp examination or Schirmer testing. Any additional ophthalmological assessments were performed as clinically indicated.

**SUPPLEMENTAL FIGURES**

**Figure S1.** CONSORT diagram.

*Objective or subjective disease progression. ^†^“Other” includes treatment discontinuation due to severe noncompliance to protocol, a development in study-specific discontinuation criteria, investigator decision, consent withdrawal, patient decision, and other (reason not stated). ^‡^Screen failure or reason for study discontinuation not stated. AI, aromatase inhibitor; CDK4/6i, cyclin-dependent kinase 4/6 inhibitor; *ESR1*m, *ESR1* mutation.

**Figure S2.** Adherence rates by treatment visit for VSAQ (A) and NEI-VFQ-25 (B).

**A.**

**B.**

Baseline was defined as the last assessment collected prior to the start of study treatment. The number of expected forms was defined as the number of patients under follow-up for patient-reported outcomes at a given timepoint. The number of evaluable forms was defined as the number of patients with questionnaires where ≥1 subscale could be determined. AI, aromatase inhibitor; CDK4/6i, cyclin-dependent kinase 4/6 inhibitor; NEI-VFQ-25, National Eye Institute Visual Function Questionnaire-25; VSAQ, Visual Symptom Assessment Questionnaire.

**Figure S3.** Incidence of patient-reported visual effects with lighting changes and degrees of bother by specific visual symptoms, as measured by the VSAQ, by treatment visit.

Data are shown from timepoints where there were data from ≥10 patients in either arm. Data are shown from patients who reported any visual effect in the past week via the questionnaire. Some totals may exceed 100% due to rounding. No patients from either arm reported being extremely bothered by overlapping shadows; shimmering, flashing, or lights; streamers, strings, or floaters; and difficulty adapting to bright or dim light while on study treatment. AI, aromatase inhibitor; CDK4/6i, cyclin-dependent kinase 4/6 inhibitor; VSAQ, Visual Symptom Assessment Questionnaire.

**Figure S4.** Responses to additional NEI-VFQ-25 items, by treatment visit.

The scores range from 0 to 100, with a higher score representing better functioning. AI, aromatase inhibitor; BL, baseline; CDK4/6i, cyclin-dependent kinase 4/6 inhibitor; NEI-VFQ-25, National Eye Institute Visual Function Questionnaire-25; SD, standard deviation; W, week.
